# Qiliqiangxin Capsule Modulates Calcium Transients and Calcium Sparks in Human Induced Pluripotent Stem Cell-Derived Cardiomyocytes

**DOI:** 10.1155/2022/9361077

**Published:** 2022-08-30

**Authors:** Yuxin Li, Zhang Zhang, Xuezeng Hao, Tingting Yu, Sen Li

**Affiliations:** ^1^School of Life Sciences, Beijing University of Chinese Medicine, Beijing, China; ^2^Dongzhimen Hospital of Beijing University of Chinese Medicine, Beijing, China

## Abstract

**Background:**

The therapeutic effects of Qiliqiangxin capsule (QLQX), a Chinese patent medicine, in patients with chronic heart failure are well established. However, whether QLQX modulates cardiac calcium (Ca^2+^) signals, which are crucial for the heart function, remains unclear. *Aim of the Study*. This study aimed to evaluate the role of QLQX in modulating Ca^2+^ signals in human induced pluripotent stem cell-derived cardiomyocytes (hiPSC-CMs).

**Materials and Methods:**

Fluorescence imaging was used to monitor Ca^2+^ signals in the cytosol and nuclei of hiPSC-CMs. For Ca^2+^ spark measurements, the line-scan mode of a confocal microscope was used.

**Results:**

The QLQX treatment substantially decreased the frequency of spontaneous Ca^2+^ transients, whereas the amplitude of Ca^2+^ transients elicited by electrical stimulation did not change. QLQX increased the Ca^2+^ spark frequency in both the cytosol and nuclei without changing the sarcoplasmic reticulum Ca^2+^ content. Interestingly, QLQX ameliorated abnormal Ca^2+^ transients in CMs differentiated from hiPSCs derived from patients with long-QT syndrome.

**Conclusions:**

Our findings provide the first line of evidence that QLQX directly modulates cardiac Ca^2+^ signals in a human cardiomyocyte model.

## 1. Introduction

Calcium is an important second messenger that participates in several physiological processes [1, 2]. In cardiomyocytes, extracellular calcium ion influx during action potentials promotes calcium release from the sarcoplasmic reticulum (SR) to the cytosol. This phenomenon is called calcium-induced calcium release and is essential for cardiac contraction [3]. Calcium in the cytoplasm flows into the nucleus through the nuclear pore complex (NPC), which regulates gene transcription. Many drugs modulate myocardial function through calcium signaling. For example, stachydrine hydrochloride targets calcium-handling proteins such as ryanodine receptors (RyRs) and phospholamban (PLN) [4]. Shengmai powder, a traditional Chinese medicine, regulates calcium signals to treat heart failure and angina pectoris [5].

The Qiliqiangxin capsule (QLQX) is a Chinese patent medicine composed of 11 herbs, including *Astragalus membranaceus*, ginseng, and *Aconitum carmichaelii* [6, 7]. Several clinical trials have shown that QLQX improves cardiac function and reduces mortality in patients with chronic heart failure (CHF) [6, 8, 9]. In rats, QLQX ameliorates cardiac remodeling caused by myocardial infarction [7, 10]. However, the effect of QLQX on cardiac calcium signals, which is crucial for heart function, remains unclear. Moreover, the recent mechanistic studies of QLQX are mainly based on animal models or animal cells. Because of species differences, experimental results in animal models cannot fully reflect the action of drugs in humans [11]. Human induced pluripotent stem cell-derived cardiomyocytes (hiPSC-CMs) have been used as a source of human cardiomyocytes for drug testing, and normal (without disease modeling) hiPSC-CMs can be used to investigate the mechanism of action of drugs [12]. Therefore, we used hiPSC-CMs to study the effect of QLQX on cardiac cytosolic and nuclear calcium signals.

## 2. Methods

### 2.1. Cells and Reagents

Normal and long-QT (KCNQ1, exon4, c.656 *G* > *A*) hiPSC-CMs were obtained from HELP Therapeutics (Nanjing, China) and cultured as described previously [13]. QLQX was obtained from Shijiazhuang Yiling Pharmaceutical Co., Ltd. (Hebei, China). According to the “Chinese Pharmacopoeia” and the relevant standards of the National Medical Products Administration, the quality standard of the QLQX capsules is stated as follows: HPLC determination of *Astragalus* by astragaloside IV (C41H68O14) shall be no less than 0.12 mg. The prescription is Astragalus 450 g, ginseng 225 g, Heishun tablets 112.5 g, Salvia 225 g, Tinglizi 150 g, Alisma 225 g, Polygonatum odoratum 75 g, cinnamon 90 g, safflower 90 g, fragrant Jiapi 180 g, and tangerine peel 75 g. The Tyrode solution contained 140 mM NaCl, 5 mM KCl, 1 mM MgCl_2_, 1 mM CaCl_2_, and 10 mM D-glucose, buffered with 10 mM HEPES at pH 7.4.

### 2.2. Cell Counting Kit-8 Assay (CCK-8)

The viability of hiPSC-CMs treated with various QLQX concentrations were examined using a CCK-8kit (Melone, China) by following the manufacturer's protocol. A plate reader (SpectraMax i3X, Molecular Devices, CA, USA) was used to measure absorbance at 450 nm.

### 2.3. Flow Cytometry

The hiPSC-CMs were fixed in 4% paraformaldehyde (PFA) at room temperature for 15 min, and the cell membranes were permeabilized with 90% precooled methanol for 30 min on ice. The permeabilized cells were incubated with the cTnT primary antibody (Abcam, UK) and an appropriate secondary antibody. A CytoFLEX flow cytometer was used to analyze the percentage of cTnT-positive cells.

### 2.4. Immunostaining

The hiPSC-CMs were fixed in 4% PFA for 20 min and then permeabilized with 0.2% Triton X-100 for 10 min. After blocking with 1% bovine serum albumin (BSA)-phosphate buffered saline (PBS) for 15 min, the samples were incubated with the cTnT antibody (Abcam) overnight at 4°C. The Alexa Fluor-555 anti-mouse IgG (Invitrogen, Carlsbad, CA, USA) was used as the secondary antibody, and the nuclei were labeled with DAPI (Solarbio, China). A confocal microscope (FV3000; Olympus, Japan) was used for imaging. We used an ER-tracker to examine the structure of the nucleoplasmic reticulum (NR) as previously described [14]. The nuclei of living cells were labeled with Hoechst 33342 and images were captured using a confocal microscope.

### 2.5. Calcium Imaging

Fluorescence imaging of Fluo-4 (Invitrogen) was used to monitor calcium signals in the cytoplasm and nucleus. The cells were incubated in a Tyrode solution containing 5 *μ*M Fluo-4-AM at 37°C for 30 min. The samples were then transferred to an inverted microscope. For local calcium signals, the confocal line-scan mode was employed, and the scanning lines were placed crossing the long axis of the cell so that the nucleus was approximately in the middle of the scanning line. Sparkmaster [15] and Peakcaller [16] were used to analyze the calcium signals.

### 2.6. High-Performance Liquid Chromatography Tandem Mass Spectrometry (HPLC-MS/MS)

To identify the compounds in QLQX using HPLC-MS/MS, QLQX was centrifuged at 12000 rpm for 10 min at 4°C, and the supernatant was diluted 2–100 times. The internal standard was passed through a 0.22 *μ*m PTPE filter, and metabolite quantitative analysis was performed. The analysis platform (Ultimate 3000LC, *Q* Exactive HF, Thermo Fisher Scientific, USA) was employed, and separation was achieved using a Zorbax Eclipse C18 column (1.8 *μ*m, 2.1 × 100 mm). The chromatographic separation conditions were column temperature, 30°C; flow rate, 0.3 mL/min; injection volume, 2 *μ*L; and autosampler temperature, 4°C. Gradient elution with mobile phase compositions A (water + 0.1% formic acid) and B (acetonitrile) was performed. Data analysis was performed using the Compound Discoverer 3.2 software.

### 2.7. Statistical Analysis

Statistical tests were performed using the SPSS software, and statistical significance was set at *P* < 0.05. All data are expressed as the mean ± standard error (SE).

## 3. Results

**Figure 1:**
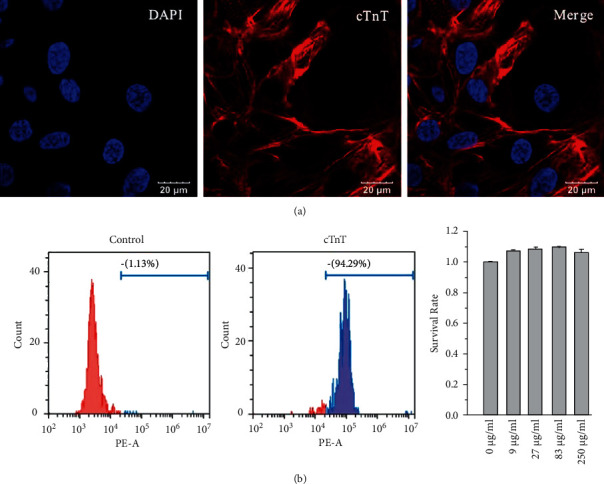
The expression of cTnT was examined by immunostaining (a) and flow cytometry analysis (b). Various concentrations of QLQX did not alter cell survival rate as examined by CCK-8 (c).

Cardiomyocytes derived from hiPSCs stained positive for cardiac troponin *T* (cTnT), a cardiac-specific protein (Figure 1(a)). The flow cytometry analysis also indicated that 94.29% of hiPSC-CMs expressed cTnT (Figure 1(b)). hiPSC-CM viability was not substantially altered by treatment with QLQX at a range of concentrations (9, 27, 83, and 250 *μ*g/ml), indicating that QLQX was not toxic to cardiomyocytes at the examined concentrations (Figure 1(c)).

**Figure 2:**
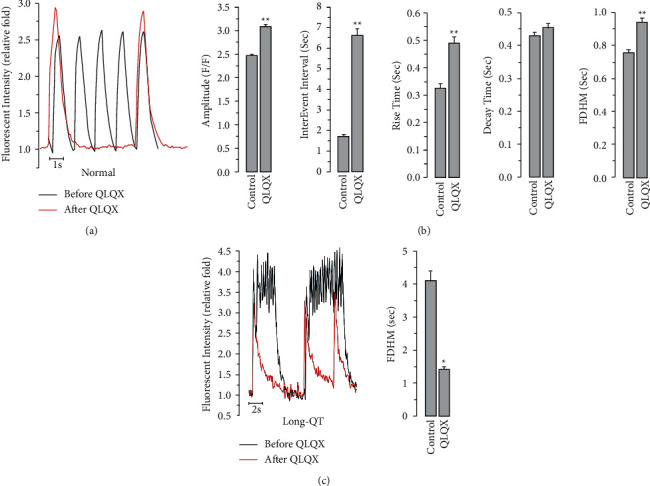
The effects of QLQX on spontaneous Ca^2+^ transients in hiPSC-CMs. (a) Representative Ca^2+^ transients before and after the QLQX application (83 *μ*g/ml, 3 minutes). (b) The QLQX treatment significantly decreased the frequency but increased the amplitude, rise time, and full duration at half maximum (FDHM) of spontaneous Ca^2+^ transients. *n* = 48–50 cells, ^*∗∗*^*P* < 0.01. (c) In long-QT hiPSC-CMs, QLQX attenuated abnormal Ca^2+^ transients. *n* = 11 cells, ^*∗∗*^*P* < 0.01.

**Figure 3:**
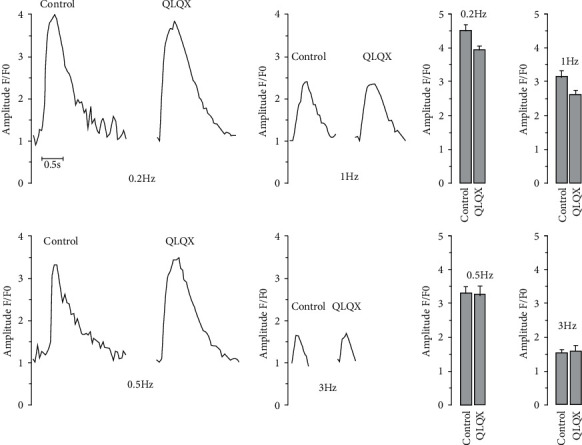
Electrical stimulation (0.2–3 Hz)-elicited Ca^2+^ transients were not affected by the QLQX treatment (83 *μ*g/ml, 3 minutes) in hiPSC-CMs. *n* = 27–30 cells.

**Figure 4:**
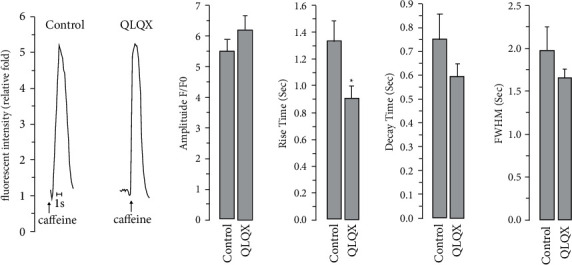
QLQX pretreatment (83 *μ*g/ml, minutes) decreased the rise time but not amplitude of caffeine (25 mM)-induced Ca^2+^ rise in hiPSC-CMs. Caffeine application was shown by arrow. *n* = 22–27 cells, ^*∗*^*P* < 0.05.

**Figure 5:**
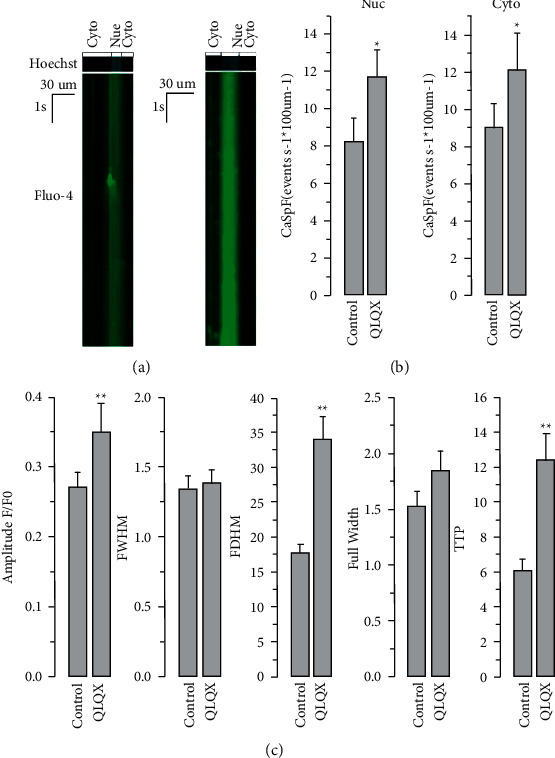
QLQX pretreatment (83 *μ*g/ml, 3 minutes) significantly increased Ca^2+^ spark frequency (CaSpF) in both the cytosol and nuclei of hiPSC-CMs (a). Ca^2+^ spark parameters, including full duration at half maximum (FDHM) and time to peak (TTP) were modulated by QLQX (b). *n* = 125 sparks from 20 cells, ^*∗*^*P* < 0.05, ^*∗∗*^*P* < 0.01. Nifedipine (3 *μ*M) was used to inhibit spontaneous Ca^2+^ transients.

**Figure 6:**
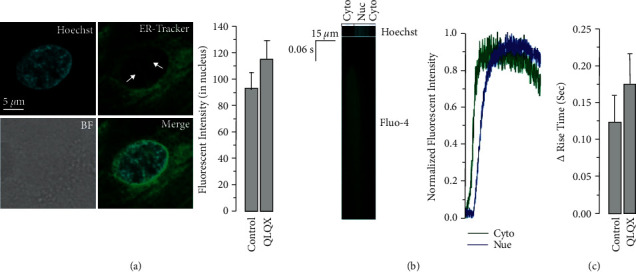
QLQX treatment (83 *μ*g/ml, 3 minutes) did not change the nucleoplasmic reticulum (NR) structure in hiPSC-CMs (a). *n* = 18–25 cells. Nuclear Ca^2+^ waves had delayed kinetics compared to that of cytosolic Ca^2+^ waves (b), which was not altered by the QLQX pretreatment in hiPSC-CMs (c). *n* = 15–20 cells. 10 mM external Ca^2+^ was used to promote Ca^2+^ waves.

As shown in Figure 2(a), the frequency of spontaneous Ca^2+^ transients was remarkably decreased by the QLQX treatment in hiPSC-CMs, together with elevated calcium transient amplitude, rise time, and full duration at half maximum (Figure 2(b)). In CMs differentiated from hiPSCs derived from patients with long-QT syndrome, abnormal Ca^2+^ transients could be observed, which were presumably caused by long trains of early afterdepolarizations [17]. QLQX effectively attenuated Ca^2+^signal abnormalities (Figure 2(c)). Next, we investigated the potential role of QLQX in modulating electrical stimulation-elicited Ca^2+^ transients in normal hiPSC-CMs. The results indicated that the amplitude of these Ca^2+^ transients was not significantly changed by the QLQX application (Figure 3). We then studied the effect of QLQX on SR Ca^2+^ content in hiPSC-CMs, and the results suggested a similar amplitude but substantially decreased rise time of caffeine-induced Ca^2+^ rise, indicating an unchanged SR Ca^2+^ content but increased RyR activity (Figure 4). Consistently, Ca^2+^ spark measurements using confocal microscopy in line scanning mode indicated that QLQX increased the Ca^2+^ spark frequency in both the cytosol and nuclei (Figure 5). As the structural basis of nuclear Ca^2+^ sparks, the NR of hiPSC-CMs revealed by the ER-tracker loading, was not substantially changed by QLQX (Figure 6(a)). Moreover, nuclear Ca^2+^ waves in hiPSC-CMs displayed delayed kinetics compared to those in the cytosol, which were also not altered by the QLQX treatment (Figure 6(b)).

To better understand the mechanisms of action of QLQX, its chemical composition was analyzed using HPLC-MS/MS, and 62 components, such as cinnamic acid, flavonoids, astragalus, coumarin, and its derivatives were identified (Supplementary Table 1).

## 4. Discussion

The therapeutic effects of QLQX have been observed in several clinical studies [18–20]. In CHF patients, the combined use of the QLQX capsules with standard treatment further reduces the level of N-terminal pro-B-type natriuretic peptide (NT-proBNP) [9], a marker of heart failure status [21]. A meta-analysis of 129 clinical trials indicated that QLQX combined with conventional treatment reduced the occurrence of major cardiovascular events and rehospitalization rates. Moreover, improvement in myocardial function without serious adverse events has been observed [20]. Mechanistic studies of QLQX have mainly been based on animal models. For example, QLQX ameliorates ventricular remodeling and improves cardiac function in a zebrafish heart failure model [22]. Furthermore, QLQX protected mice against damage caused by myocardial infarction induced by left coronary artery ligation. *In vitro* experiments have also shown that QLQX attenuates hypoxia-induced apoptosis in cardiomyocytes [23]. In normal and hypertrophic rat cardiomyocytes, QLQX inhibited *I*_Ca,L_, which may serve as one of the underlying mechanisms of its therapeutic effects [24]. A recent study investigated the protective effect of QLQX on doxorubicin-induced cardiotoxicity in a rat model [25]. Left ventricular remodeling is also alleviated by QLQX in rats with pressure overload-induced heart failure [26].

hiPSC-CMs have been widely used as a source of human cardiomyocytes for evaluating the effects of drugs on cardiac function. For example, field stimulation-induced contraction and Ca^2+^ transients of hiPSC-CMs have been employed as key indicators of drug-induced changes in myocardial function [12]. hiPSC-CMs have also been used to investigate the role of ginsenoside Rb1 in ameliorating aconitine-induced cardiotoxicity by regulating calcium homeostasis [27]. Moreover, three-dimensional engineered heart tissues constructed with 10 normal hiPSC cell-line-differentiated CMs have been used to detect the positive inotropic effects of seven drugs under electrical stimulation [28]. Another study employed human ventricular-like cardiac tissue strips (hvCTS) and organoid chambers (hvCOCs) constructed with disease-free hiPSC-CMs to screen 25 cardioactive chemicals covering different drug categories [29]. Therefore, hiPSC-CMs without disease modeling are suitable for drug evaluation, and we used this model to comprehensively examine the role of QLQX in modulating cardiac Ca^2+^ signals.

We first observed that spontaneous Ca^2+^ transient frequency, an indicator of the beating rate, was remarkably decreased by QLQX in hiPSC-CMs (Figure 2). The hiPSC-CMs used were a mixture of ventricular, atrial, and pacemaker-like cells. hiPSC-CMs also express pacemaker currents (*I*_*f*_), which are absent in human adult CMs, leading to the innate automaticity of hiPSC-CMs [30]. Therefore, the spontaneous beating rate of hiPSC-CMs has been widely used to examine the chronotropic effects of drugs. Epidemiological studies have shown that elevated heart rate is a risk factor for cardiovascular disease in healthy people [31]; an increase in heart rate by 10 beats per minute is correlated with a 20% elevated risk of cardiac death [32]. The heart rate-lowering medications can reduce mortality [33]. For example, ivabradine attenuates heart failure by reducing the heart rate of patients [33]. In addition, *β*-blockers, as commonly prescribed drugs for the treatment of cardiovascular diseases, decrease heart rate to limit myocardial oxygen consumption, thereby improving heart function [34]. Some traditional Chinese medicines, such as ginsenoside, one of the active ingredients of ginseng, reduce heart rate, thereby alleviating cardiac dysfunction and remodeling in heart failure [35].

Ca^2+^ signals reflect the underlying mechanisms of altered cardiac contractile properties during drug treatment. Thus, we comprehensively measured the effect of QLQX on calcium homeostasis in hiPSC-CMs and found that QLQX increased the amplitude of spontaneous Ca^2+^ transients (Figure 2(a) and 2(b)), which was presumably due to its negative chronotropic effect because hiPSC-CMs harbored a negative force-frequency response (i.e., lowering beating frequency would lead to higher Ca^2+^ transient amplitude). This hypothesis was supported by our observation that QLQX did not change the amplitudes of Ca^2+^ transients elicited by the 0.2–3 Hz electrical stimulation (Figure 3). As localized calcium signals, calcium sparks reflect the open probability of the RyRs. Indeed, low concentrations of caffeine, an activator of the RyRs, can increase calcium spark frequency [36]. Our results suggest that the QLQX application substantially increased Ca^2+^ spark frequency in both the cytosol and nuclei (Figure 5). Furthermore, the elevated amplitude and duration of Ca^2+^ sparks after the QLQX treatment indicated stronger Ca^2+^ release. One potential drawback of the increased calcium spark frequency is the elevated level of Ca^2+^ leakage from the SR. However, QLQX did not change the SR Ca^2+^ content of hiPSC-CMs, as determined by the application of a high concentration of caffeine (Figure 4). These results suggest that QLQX may target multiple Ca^2+^-handling proteins to regulate Ca^2+^ homeostasis in hiPSC-CMs.

**Figure 7:**
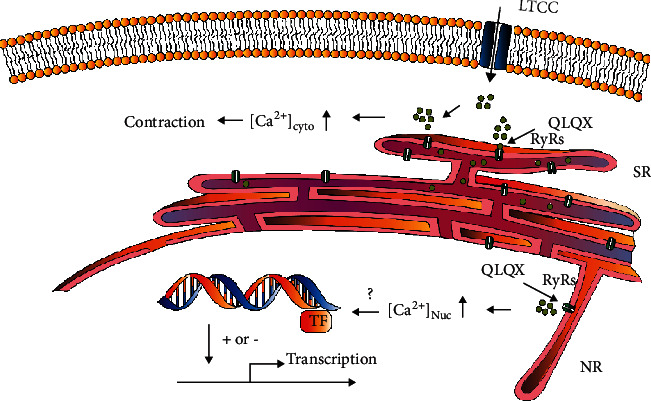
Diagram showing that QLQX increased cytosolic/nuclear Ca^2+^ sparks and may potentially modulate nuclear Ca^2+^-mediated gene transcription.

In cardiomyocytes, Ca^2+^ exhibits different functions in different subcellular locations. In the cytoplasm, the major function of Ca^2+^ is to regulate cardiac contraction through EC coupling. In the nucleus, Ca^2+^ regulates gene transcription, and the activation of nuclear Ca^2+^-dependent transcription factors is a part of the process called excitation-transcription coupling (ETC), which is the link between extracellular signals and cardiomyocyte reprogramming [37]. For example, endothelin 1 induces inositol-1,4,5-trisphosphate receptor (IP_3_R)-mediated local nuclear Ca^2+^ release, which in turn activates nuclear CaMKII and affects downstream gene transcription during cardiac hypertrophy [38]. Therefore, nuclear Ca^2+^ plays an important role in regulating physiological functions of cardiomyocytes [39]. As mentioned above, our results indicated that QLQX increased the nuclear Ca^2+^ spark frequency; however, the effect of nuclear calcium activation on gene transcription warrants further investigation (Figure 7).

Our HPLC-MS/MS results showed that QLQX contains numerous cardioprotective chemical components. For example, choline can regulate the expression of key calcium-handling proteins, such as STIM1 and Orai1 and attenuate the angiotensin II-induced elevation of intracellular calcium, thereby alleviating the cardiac remodeling induced by abdominal aorta coarctation in rats [40]. As another constituent of QLQX, stachydrine inhibits SR calcium leakage and improves the calcium transient amplitude and cardiac function in mice with transverse aorta constriction [4]. Moreover, caffeic acid regulates calcium and potassium channels and decreases the heart rate, showing a protective effect on cardiovascular diseases [41]. Therefore, many chemical components of QLQX are closely related to the regulation of cardiac calcium signaling, and the observations in our study may reflect the multitarget pharmacological mechanisms of QLQX.

## 5. Conclusion

The current study systematically investigated the effect of QLQX on cardiac Ca^2+^ signals using a human cardiomyocyte model, and the results suggest that QLQX could reduce the spontaneous beating frequency of hiPSC-CMs, as reflected by Ca^2+^ transient measurements. Moreover, QLQX substantially promoted the generation of Ca^2+^ sparks in both the cytosol and nucleus. Taken together, these findings provide the first line of evidence that QLQX directly modulates cardiac Ca^2+^ signals in human cardiomyocytes, which may lead to a better understanding of the mechanism of action of QLQX and contribute to the development of novel modern Chinese drugs.

## Data Availability

The data used to support the findings of this study are included within the article.

## Supplementary Materials

Supplementary Materials Supplementary Table 1: The identified compounds in QLQX by HPLC-MS/MS.
