# Supplementary material for: Qiliqiangxin Capsule Modulates Calcium Transients and Calcium Sparks in Human Induced Pluripotent Stem Cell-Derived Cardiomyocytes
Source: Evid Based Complement Alternat Med. 2022 Aug 30;2022:9361077. doi: 10.1155/2022/9361077 (PMC9448542; doi:10.1155/2022/9361077)
Supplement: Supplementary Materials — Supplementary Table 1: The identified compounds in QLQX by HPLC-MS/MS. [file 9361077.f1.docx]

Supplementary Table 1. The identified compounds in QLQX by HPLC-MS/MS.

| NO. | RT [min] | Annotation MW | Calc. MW | Molecular Formula | Proposed Compound | CAS_num | Class |
| --- | --- | --- | --- | --- | --- | --- | --- |
| 1 | 0.724 | 174.11168 | 174.11179 | C_6_H_14_N_4_O_2_ | DL-Arginine | 7200-25-1 | Carboxylic acids and derivatives |
| 2 | 0.748 | 103.09971 | 103.10018 | C_5_H_13_NO | Choline | 62-49-7 | Organonitrogen compounds |
| 3 | 0.763 | 295.09167 | 295.09022 | C_11_H_13_N_5_O_5_ | Azidamfenicol | 13838-08-9 | Carboxylic acids and derivatives |
| 4 | 0.783 | 117.07898 | 117.07929 | C_5_H_11_NO_2_ | Betaine | 107-43-7 | Carboxylic acids and derivatives |
| 5 | 0.792 | 162.05282 | 162.05287 | C_6_H_10_O_5_ | DiethylpyrocarbonateC | 1609-47-8 | Organic carbonic acids and derivatives |
| 6 | 0.793 | 137.04768 | 137.04781 | C_7_H_7_NO_2_ | Methyl isonicotinate | 2459-09-8 | Pyridines and derivatives |
| 7 | 0.795 | 115.06333 | 115.06362 | C_5_H_9_NO_2_ | D-(+)-Proline | 344-25-2 | Carboxylic acids and derivatives |
| 8 | 0.798 | 126.03169 | 126.03189 | C_6_H_6_O_3_ | Pyrogallol | 87-66-1 | Phenols |
| 9 | 0.81 | 474.15625 | 474.15819 | C_20_H_24_Cl_2_N_10_ | Picloxydine | 5636-92-0 | Benzene and substituted derivatives |
| 10 | 0.813 | 257.10282 | 257.10286 | C_8_H_20_NO_6_P | Choline Alfoscerate | 28319-77-9 | Glycerophospholipids |
| 11 | 0.817 | 143.09463 | 143.09468 | C_7_H_13_NO_2_ | DL-Stachydrine | 4136-37-2 | Carboxylic acids and derivatives |
| 12 | 0.837 | 129.07898 | 129.07912 | C_6_H_11_NO_2_ | D-(+)-Pipecolinic acid | 1723-00-8 | Carboxylic acids and derivatives |
| 13 | 0.841 | 244.06954 | 244.06917 | C_9_H_12_N_2_O_6_ | Pseudouridine | 1445-07-4 | Nucleoside and nucleotide analogues |
| 14 | 0.89 | 192.027 | 192.02624 | C_6_H_8_O_7_ | Citric acid | 77-92-9 | Carboxylic acids and derivatives |
| 15 | 0.93 | 129.04259 | 129.04274 | C_5_H_7_NO_3_ | L-Pyroglutamic acid | 98-79-3 | Carboxylic acids and derivatives |
| 16 | 1.112 | 123.03203 | 123.03223 | C_6_H_5_NO_2_ | Nicotinic acid | 59-67-6 | Pyridines and derivatives |
| 17 | 1.113 | 135.0545 | 135.05458 | C_5_H_5_N_5_ | Adenine | 73-24-5 | Imidazopyrimidines |
| 18 | 1.144 | 179.09463 | 179.09477 | C_10_H_13_NO_2_ | Salsolinol | 525-72-4 | Tetrahydroisoquinolines |
| 19 | 1.188 | 189.06372 | 189.06296 | C_7_H_11_NO_5_ | N-Acetylglutamic acid | 1188-37-0 | Carboxylic acids and derivatives |
| 20 | 1.19 | 267.09675 | 267.09664 | C_10_H_13_N_5_O_4_ | Adenosine | 58-61-7 | Purine nucleosides |
| 21 | 1.279 | 111.07965 | 111.08003 | C_5_H_9_N_3_ | Histamine | 51-45-6 | Organonitrogen compounds |
| 22 | 1.291 | 151.04941 | 151.04956 | C_5_H_5_N_5_O | Guanine | 73-40-5 | Imidazopyrimidines |
| 23 | 1.292 | 283.09167 | 283.09172 | C_10_H_13_N_5_O_5_ | Crotonoside | 1818-71-9 | Purine nucleosides |
| 24 | 1.317 | 283.09167 | 283.09157 | C_10_H_13_N_5_O_5_ | Guanosine | 118-00-3 | Purine nucleosides |
| 25 | 1.853 | 198.10044 | 198.10044 | C_9_H_14_N_2_O_3_ | Metharbital | 50-11-3 | Diazines |
| 26 | 2.974 | 198.05282 | 198.0522 | C_9_H_10_O_5_ | Danshensu | 76822-21-4 | Phenylpropanoic acids |
| 27 | 4.473 | 117.05785 | 117.05806 | C_8_H_7_N | Indole | 120-72-9 | Indoles and derivatives |
| 28 | 5.381 | 162.03169 | 162.03176 | C_9_H_6_O_3_ | 7-Hydroxycoumarine | 93-35-6 | Coumarins and derivatives |
| 29 | 5.628 | 250.08412 | 250.08409 | C_13_H_14_O_5_ | Citrinin | 518-75-2 | Benzopyrans |
| 30 | 5.628 | 309.15762 | 309.15755 | C_16_H_23_NO_5_ | Sinapine | 18696-26-9 | Cinnamic acids and derivatives |
| 31 | 5.69 | 180.04226 | 180.0415 | C_9_H_8_O_4_ | Caffeic acid | 331-39-5 | Cinnamic acids and derivatives |
| 32 | 5.767 | 211.08446 | 211.08397 | C_10_H_13_NO_4_ | Methoxytyrosine | 7636-26-2 | Carboxylic acids and derivatives |
| 33 | 6.512 | 446.1213 | 446.12102 | C_22_H_22_O_10_ | Glycitin | 40246-10-4 | Isoflavonoids |
| 34 | 6.766 | 538.11113 | 538.11128 | C_27_H_22_O_12_ | Lithospermic acid | 28831-65-4 | 2-arylbenzofuran flavonoids |
| 35 | 6.883 | 192.04226 | 192.04159 | C_10_H_8_O_4_ | Scopoletin | 92-61-5 | Coumarins and derivatives |
| 36 | 6.894 | 580.17921 | 580.17946 | C_27_H_32_O_14_ | Naringin | 10236-47-2 | Flavonoids |
| 37 | 7.154 | 610.18977 | 610.1902 | C_28_H_34_O_15_ | Hesperidin | 520-26-3 | Flavonoids |
| 38 | 7.362 | 589.2887 | 589.28862 | C_31_H_43_NO_10_ | Benzoylmesaconine | 63238-67-5 | Prenol lipids |
| 39 | 7.43 | 440.36543 | 440.36531 | C_30_H_48_O_2_ | Roburic acid | 6812-81-3 | Prenol lipids |
| 40 | 7.449 | 180.04226 | 180.04232 | C_9_H_8_O_4_ | Caffeic acid | 331-39-5 | Cinnamic acids and derivatives |
| 41 | 7.45 | 360.08452 | 360.08449 | C_18_H_16_O_8_ | Rosmarinic acid | 20283-92-5 | Cinnamic acids and derivatives |
| 42 | 7.475 | 188.10486 | 188.10411 | C_9_H_16_O_4_ | Azelaic acid | 123-99-9 | Fatty Acyls |
| 43 | 7.738 | 538.11113 | 538.11113 | C_27_H_22_O_12_ | Lithospermic acid | 28831-65-4 | 2-arylbenzofuran flavonoids |
| 44 | 7.756 | 494.1213 | 494.12129 | C_26_H_22_O_10_ | Salvianolic acid A | 96574-01-5 | Stilbenes |
| 45 | 7.776 | 430.12638 | 430.12624 | C_22_H_22_O_9_ | Ononin | 486-62-4 | Isoflavonoids |
| 46 | 8.834 | 168.04226 | 168.04225 | C_8_H_8_O_4_ | 4-Methoxysalicylic acid | 2237-36-7 | Benzene and substituted derivatives |
| 47 | 8.89 | 284.06847 | 284.06832 | C_16_H_12_O_5_ | 5-O-Methylgenistein | 4569-98-6 | Isoflavonoids |
| 48 | 8.897 | 284.06847 | 284.06834 | C_16_H_12_O_5_ | Glycitein | 40957-83-3 | Isoflavonoids |
| 49 | 9.099 | 422.19407 | 422.19429 | C_22_H_30_O_8_ | Valtrate | 18296-44-1 | Prenol lipids |
| 50 | 9.231 | 167.05824 | 167.05832 | C_8_H_9_NO_3_ | Pyridoxal | 66-72-8 | Pyridines and derivatives |
| 51 | 9.354 | 424.37052 | 424.37008 | C_30_H_48_O | Lupenone | 1617-70-5 | Prenol lipids |
| 52 | 9.574 | 328.22497 | 328.22502 | C_18_H_32_O_5_ | Corchorifatty acid F | 95341-44-9 | Fatty Acyls |
| 53 | 9.629 | 956.49808 | 956.49843 | C_48_H_76_O_19_ | Ginsenoside Ro | 34367-04-9 | Prenol lipids |
| 54 | 10.305 | 454.3447 | 454.34426 | C_30_H_46_O_3_ | Dehydrotrametenolic acid | 29220-16-4 | Prenol lipids |
| 55 | 10.426 | 268.07356 | 268.07338 | C_16_H_12_O_4_ | Formononetin | 485-72-3 | Isoflavonoids |
| 56 | 11.113 | 402.13147 | 402.131 | C_21_H_22_O_8_ | Nobiletin | 478-01-3 | Flavonoids |
| 57 | 13.187 | 203.13101 | 203.13083 | C_13_H_17_NO | Crotamiton | 483-63-6 | Benzene and substituted derivatives |
| 58 | 14.894 | 266.15518 | 266.15504 | C_12_H_26_O_4_S | Dodecyl sulfate | 151-41-7 | Organic sulfuric acids and derivatives |
| 59 | 18.121 | 256.24023 | 256.23987 | C_16_H_32_O_2_ | Palmitic acid | 57-10-3 | Fatty Acyls |
| 60 | 19.211 | 132.04226 | 132.04254 | C_5_H_8_O_4_ | Ethylmalonic acid | 601-75-2 | Fatty Acyls |
| 61 | 19.221 | 162.05282 | 162.05315 | C_6_H_10_O_5_ | Diethylpyrocarbonate | 1609-47-8 | Organic carbonic acids and derivatives |
| 62 | 19.526 | 158.05791 | 158.0582 | C_7_H_10_O_4_ | Ethyl acetonoxalate | 615-79-2 | Keto acids and derivatives |
